# Detection of a rare AXIN2 variant in an Iranian family with hypodontia and oligodontia

**DOI:** 10.34172/joddd.2022.018

**Published:** 2022-10-15

**Authors:** Shiva Safari, Asghar Ebadifar, Hossien Najmabadi, Koorosh Kamali, Seyedeh Sedigheh Abedini, Mohammad Mousavi

**Affiliations:** ^1^Orthodontist, Private Practice, Tehran, Iran; ^2^Dentofacial Deformities Research Center, Research Institute of Dental Sciences, Department of Orthodontic, Faculty of Dentistry, Shahid Beheshti University of Medical Sciences, Tehran, Iran; ^3^Genetic Research Centre, University of Social Welfare and Rehabilitation Sciences, Tehran, Iran; ^4^Department of Public Health, School of Public Health, Zanjan University of Medical Sciences, Zanjan, Iran; ^5^Dentofacial Deformities Research Center, Research Institute of Dental Sciences, Shahid Beheshti University of Medical Sciences, Tehran, Iran

**Keywords:** PAX9, MSX1, AXIN2, Oligodontia

## Abstract

**Background.** Hypodontia, or the absence of one or more teeth during tooth formation, is a highly prevalent dental anomaly. Nevertheless, the main causes are still unknown. Mutations in *PAX9*, *MSX1*, *WNT10A*, and *AXIN2* genes are most commonly associated with non-syndromic tooth agenesis in the literature. This study investigated these candidate genes in an Iranian family with non-syndromic hypodontia and oligodontia.

**Methods.** Peripheral blood samples of the proband and her family members were collected, and DNA extractions using the salting-out method were carried out. In addition, polymerase chain reaction (PCR) and Sanger sequencing for candidate genes were performed.

**Results.** A missense variant (rs4904210) was identified in the *PAX9* gene, with one heterozygous missense variant (rs2240308) and one stop-gained variant (rs121908568) in the *AXIN2* gene.

**Conclusion.** By surveying similar studies and analyzing the variant in bioinformatics websites, we concluded that the heterozygous stop-gained variant rs121908568 in exon 8 of the *AXIN2* gene could be responsible for tooth agenesis in the Iranian population.

## Introduction

 Tooth agenesis is a common dental anomaly in the normal population.^1-4^ It is mostly classified based on the number of affected teeth. Hypodontia is defined as the developmental absence of 1‒6 permanent teeth.^2^ The term oligodontia refers to the congenital missing of ≥6 teeth, excluding third molars, and anodontia represents the clinical situation of complete failure of the dentition.^5^ Tooth agenesis affects the quality of life by causing aesthetics, masticatory, and speech problems.^1^ The prevalence of hypodontia has been reported to be 6.4% worldwide. Mandibular second premolars are the most affected teeth, followed by maxillary lateral incisors and second premolars.^6^ The prevalence of tooth agenesis in the Iranian population has been reported at 10.9%, and mandibular second premolars are the most affected.^7^

 Tooth development starts in the second month of embryogenesis and is regulated by genetic networks and tissue interactions like other ectodermal organs.^5^ Although the absence of teeth can result from environmental factors, the cause of this disease is often genetic aberrations because the position, number, size, and shape of the teeth are strongly influenced by genetics.^8^ Studies on monozygotic twins have confirmed that genetics plays a critical role in congenital tooth agenesis.^9^ It has been suggested that isolated tooth agenesis can result from mutations in *MSX1*, *PAX9*, *WNT10A*, *AXIN2*, *EDA*, *KDF1*, and *SMOC2* genes in autosomal and X-linked traits.^10-14^ Transcription factor genes *MSX1* and *PAX9* were the first identified genes responsible for tooth agenesis.^15^

*MSX1* of the Homeobox family is the first reported gene associated with tooth agenesis. Over 20 mutations in the *MSX1* gene have been identified to cause isolated tooth agenesis.^16^

*PAX9* gene is essential for the development of teeth and several organs. Along with *MSX1*, *PAX9* mutations are widely surveyed in odontogenesis.^17^*MSX1* mutations mostly lead to second molar agenesis, and *PAX9* mutations cause molar agenesis.^18^* AXIN2* gene encodes a protein that regulates the stability of β-catenin, involved in the Wnt pathway. Changes in the Wnt signaling pathway can lead to tooth agenesis and predispose to cancer.^19^

*WNT10A* is expressed in dental epithelium and enamel knots during tooth formation and encodes Wnt ligands. In recent studies, *WNT10A* gene variants have been detected in up to 50% of patients with tooth agenesis.^20^ The mutations of the gene are also responsible for autosomal recessive ectodermal dysplasia and severe non-syndromic tooth agenesis.^21,22^

 In this study, we investigated four candidate genes *MSX1*, *PAX9*, *AXIN2*, and *WNT10A* in an Iranian family with hereditary non-syndromic tooth agenesis to find the variants responsible for tooth agenesis.

## Materials and Methods

###  Clinical evaluation

 The proband of the study was selected in the Department of Orthodontics, Dental School, Shahid Beheshti University of Medical Science, after confirming the familial history of hypodontia. All the participants were clinically examined by a dental professional for tooth agenesis and other craniofacial anomalies. The control subjects were selected from the Iranian database (http://www.iranome.ir/) containing 800 healthy Iranian citizens and unaffected members of the family.

###  Molecular analysis

 DNA extraction was performed on peripheral blood samples of all the participants using the salting-out method. Primers were designed for all the coding and non-coding regions of *MSX1*, *PAX9*, *AXIN2*, and *WNT10A* genes using the Primer3 program, considering the data in the UCSC genome browser (all the designed primers are available if needed). Polymerase chain reaction (PCR) reaction mix was carried out by forward and reverse primers with 10-pmol concentration and DNA (50-100 ng) in a total volume of 20 µmol using Super PCR Master Mix 2X (Yektatajhiz). Mutation screening for all the subjects was performed using direct Sanger sequencing (ABI PRISMTM3100; Applied Biosystems, Foster City, CA, USA).

 The minor allele frequency (MAF) of the reported variants was extracted from online databases such as https://asia.ensembl.org/, https://genome.ucsc.edu/, and https://www.internationalgenome.org/1000-genomes-browsers/. For the Iranian control group, the data available at http://www.iranome.ir/ was used. The prediction of the possible impact of the amino acid substitution on protein function was checked via PolyPhen-2, SIFT, and PROVEAN. The pathogenicity score of variants was determined online via https://www.ncbi.nlm.nih.gov/clinvar/, https://varsome.com/, http://wintervar.wglab.org/, and http://www.iranome.ir/ websites. All the variants were interpreted according to ACMG standards and guidelines.

## Results

###  Clinical findings

**Figure 1:**
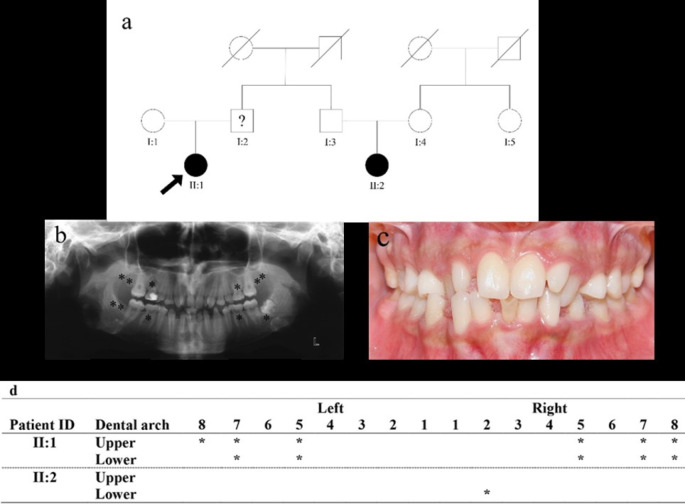
Pedigree and clinical features. (a) The pedigree of the family; the arrow indicates the proband. (b) The panoramic radiograph of the proband (black stars: missing permanent teeth). (c) The intraoral photograph of the proband. (d) The table represents the missing teeth in each quadrant.

 The proband of the family was diagnosed with oligodontia according to clinical and radiographic examinations carried out by a dental specialist (Figure 1). She had eleven missing teeth in four quadrants, including second premolars and second molars. Except for the left mandibular third molar, the other three were missing. The clinical records also showed decreased tooth size, defined as microdontia. Sweat glands, skin, and skeletal structure showed no abnormalities in the general evaluation. The father (patient: I: 2) also had missing teeth. Since the dental history was unavailable, we could not determine whether the missing teeth were due to extraction or congenitally missing. Patient (II: 2) showed isolated tooth agenesis in the mandibular second lateral incisor. The parents had complete dentition. There was no history of cancer in the family.

###  Sequencing analysis

 In the *PAX9* gene, we detected two 5´ UTR variants in intron 3: rs12883298 in heterozygous form in patient II: 2 and homozygous form in patient II: 1 and rs12882923 in homozygous form in both patients. The missense variant rs4904210 in exon 4 of patient II: 1 (homozygous) and patient II: 1 (heterozygous) was also found.

 In exon 1 of the *MSX1* gene, a heterozygous missense variant (rs36059701) was found in patient II: 1. In intron 1, the homozygous form of intronic variant rs149370601 was found in patient II: 2, and heterozygous form in patient II: 1. The 3´ UTR variant rs8670 in heterozygous form was identified in both patients.

**Figure 2:**
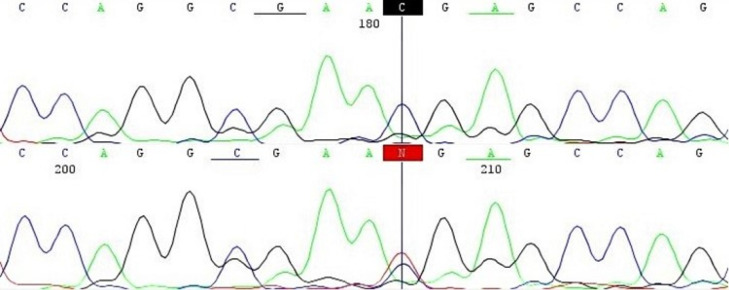
Chromatograph from sequencing of exon 8 of AXIN2 gene wild type and rs121908568.

 The sequencing of coding and non-coding areas in the *AXIN2* gene revealed a heterozygous missense variant (rs2240308) and a heterozygous synonymous variant (rs111470596) in exon 2 of patient II: 2. In intron 5, a 3´ UTR variant (rs8078753) and an intronic variant (rs11658824) were detected in homozygous form in patient II: 1 and heterozygous form in patient II: 2. Another heterozygous 3´ UTR variant (rs1422017403) was also found in patient II: 1. The exon 6 of the *AXIN2* gene of patient II: 1 and II: 2 revealed a homozygous synonymous variant (rs9915936) and another heterozygous synonymous variant (rs1133683) in patient II: 2 and in homozygous form in patient II: 1. There was also a report of a homozygous intronic variant in intron 7 of both patients II: 1 and II: 2 (rs28760438). We were also able to identify a heterozygous synonymous variant (rs143243661) in exon 8 of both patients II: 1 and II: 2, and a heterozygous stop-gained variant (rs121908568) in patient II: 1 (Figure 2).

## Discussion

 Congenital absence of teeth or tooth agenesis is a consequence of disturbances in the early stages of tooth formation. It can be part of a syndrome, such as ectodermal dysplasia, or occur as a sporadic trait with autosomal dominant, autosomal recessive, or X-linked inheritance.^23^

*MSX1* homeobox gene expression in the dental mesenchyme is critical in the early tooth formation stages.^24^ The *MSX1* encodes a DNA-binding protein, which is essential for initiating tooth development. The protein regulates gene expression and increases the rate of the transcription process.^9,10^ During initiation, bud, cap, and bell stages of ontogenesis, *PAX9* is expressed in the dental mesenchyme. *PAX9*-paired box gene cooperates with *MSX1* to facilitate the bud-to-cap stage transition. Therefore mutations causing loss of function in *MSX1* and *PAX9* in humans can lead to the partial failure of tooth development.^10^

 One missense variant (rs4904210) with a global MAF of 0.331 was found in the *PAX9 *gene. The Iranian population MAF is 0.352 for this variant.^25^ Lee et al^26^ performed a study on the Korean and Japanese populations to investigate the role of *PAX9* SNPs on tooth agenesis and morphology. Although they found no correlation between rs4904210 polymorphism and tooth agenesis, a significant association in crown size was seen. The homozygous trait of the variant could explain the reason for microdontia in patient II: 1. Zhang et al^27^ performed a meta-analysis to examine the correlation between hypodontia and *PAX9 *polymorphisms and concluded that rs4904210 has no significant association with hypodontia. Wang et al^28^ raised the possibility that structural and functional changes of protein as a result of rs4904210 variant is a risk factor for oligodontia patients in the Chinese population. A high prevalence of this variant in Iranians and the world’s normal population and its presence in normal family members lead us to the conclusion that the variant could not be the cause of hypodontia.

 Callahan et al^1^ reported that the rs2240308 variant of the *AXIN2* gene has a significant association with tooth agenesis in cases with at least one missing incisor in Brazilian–Turkish cases. However, the possible impact of the amino acid substitution on the structure and function of the *AXIN2* protein was predicted to be benign. In addition, a high MAF of the variant, both in the global and Iranian populations, reduces the possibility of an effect on tooth agenesis.

 The synonymous variant (rs143243661) is found in both II: 1 and II: 2 patients with different clinical signs. Based on the score and internal cut-off values and phenotype diversity, the rs143243661 variant is interpreted as benign for this disease. In this study, we also detected another variant in the *AXIN2* gene (rs111470596) with a very low frequency in the Iranian population (0.00125). However, since the variant is synonymous and absent in the proband, there is a low probability that it was involved in the present phenotype.^25^

 The Wnt pathway controls embryonic developmental pattern and morphogenesis of most organs, including odontogenesis.^29,30^ It has been suggested that *AXIN2* expression is a negative regulator of the Wnt signaling pathway.^1^ The rs121908568 variant in exon 8 creates a premature translational stop signal (p.Arg656*) in the *AXIN2 *gene. The variant results in the disruption of protein products and truncated *AXIN2* proteins. p.Arg656 stop mutations result in the deletion of the DIX domain of the *AXIN2* gene. DIX domains are essential for protein interactions and their ability to modulate β-catenin stability.^31^ As a result, β-catenin accumulates in nuclei and leads to the over-activation of the pathway.^32,33^This mutation is reported to induce colorectal cancer solely or along with oligodontia.^29,34^ This variant has no frequency in human databases and is detected only in the proband of the family suffering from oligodontia. Accordingly, it has been classified as pathogenic.^35^

## Conclusion

 Our findings may imply that the *AXIN2* variant rs121908568, detected on exon 8 of the proband, causes a malfunction of the AXIN2 protein by creating a premature stop codon. It is considered responsible for the patient’s clinical profile and oligodontia.

## Acknowledgments

 We are grateful to all the members of the families for their participation in the study. We would like to express our sincere appreciation for the support and help of the Genetics Research Collaboration of Shahid Beheshti University of Medical Sciences.

## Authors’ Contribution

 Study concept and design: SS and AE. Acquisition of data:SS. Analysis and interpretation of data: SS, AE, and HN. Drafting of the manuscript: SS and MM. Critical revision of the manuscript for important intellectual content: KK and SSA. Administrative, technical, and material support: SS, AE, SS, and AE. Study supervision: MM

## Funding

 The publishing of this study was supported by the Dental Research Center at Shahid Beheshti University of Medical Sciences; grant number: 855 T.

## Ethics Approval

 The Ethics Committee of Shahid Beheshti University of Medical Sciences approved this study. (Study number: IR.SBMU.DRC.REC.1398.085). Informed consent was obtained from all the participants.

## Competing Interests

 There is no conflict of interest to declare.
